# When Learning Goal Orientation Leads to Learning From Failure: The Roles of Negative Emotion Coping Orientation and Positive Grieving

**DOI:** 10.3389/fpsyg.2021.608256

**Published:** 2021-04-29

**Authors:** Wenzhou Wang, Shanghao Song, Xiaoxuan Chen, Wenlong Yuan

**Affiliations:** ^1^Department of Human Resource Management, Business School, Beijing Normal University, Beijing, China; ^2^Department of Business Administration, Asper School of Business, University of Manitoba, Winnipeg, MB, Canada

**Keywords:** learning from failure, learning goal orientation, loss orientation, restoration orientation, positive grieving

## Abstract

Considering failure is a common result in project management, how to effectively learn from failure has becoming a more and more important topic for managers. Drawing on the goal orientation theory and grief recovery theory, the purpose of this paper is to clarify the impact of learning goal orientation on learning from failure. Furthermore, this paper examines the mediating effect of two negative emotion coping orientations (restoration orientation and loss orientation) and the moderating effect of positive grieving in this relationship. The results indicated that: (1) A learning goal orientation is positively related to learning from failure; (2) As a dual-path mediation model, restoration orientation and loss orientation mediate the relationship between a learning goal orientation and learning from failure; and (3) Positive grieving negatively moderates the relationship between a loss orientation and learning from failure.

## Introduction

Failure is inevitable in today’s business environment and may bring adverse consequences to the enterprise, but failure can also bring great value and experience to the enterprise. As a result, more and more studies are focusing on failure and individual’s learning behavior after failure. Learning from failure means that **“**individuals can gain knowledge and skills from failure and can apply these knowledge and skills in practice**”** (Shepherd et al., 2011). Previous studies have shown that learning from failure can have a positive impact on individuals; this includes reducing the risk of future organizational failures (Ingram and Baum, 1997), improving innovation (Arenas et al., 2006), and improving performance (Argote and Darr, 2001). Because of the great value contained in failure, recent studies have begun to explore the antecedent variables (e.g., leadership, organizational culture, and team atmosphere) of learning from failure (Carmeli and Sheaffer, 2008) to make a better failure management for employees to meet the next challenge. However, most scholars focus their research on variables associated with specific failure events (e.g., shame, guilt; according to Wang et al., 2018), but pay scant attention to stable psychological variables such as cognition orientation and behavior patterns, which have been found to affect individuals’ learning behavior (Woolfolk, 1995). Specifically, goal orientation theory emphasizes that an individual’s goal orientation, as the cognition and understanding of the achievement, influences behavior responses (Dweck and Leggett, 1988). A learning goal orientation refers to “a tendency for individuals to the desire to develop the self by acquiring new skills, mastering new situations, and improving one’s competence” (Vandewalle and Cummings, 1997). Learning goal orientation has been shown to have a strong driving effect on the motivation of individuals to learn and master skills, and it plays an important role in promoting individuals to make positive behaviors: for example, improving work performance (Chughtai and Buckley, 2011) and promoting innovation behavior (Hirst et al., 2009). However, most of these studies are about its possible important impact on success, ignoring the important role on individual’s behavior after failure. Therefore, exploring the important role of learning goal orientation in the learning behavior of individuals after failure can analyze the psychological process that affects failure from a deeper level, so as to make up for the lack of literature on individual’s learning from failure in the past. As the R&D personnel of high-tech enterprises, as the core component of the enterprise, studying its solutions to failure has a key effect on the stability and sustainable development of the organization (Wang et al., 2013).

In order to deeply explore the role of learning goal orientation on individual’s learning from failure, we need to further explore the mediation variables that may affect this role. Goal-orientation theory believes that goal orientation will affect the individual’s cognitive or emotional tendency toward events, which in turn will trigger behavioral responses (Dweck and Leggett, 1988). In fact, many scholars associate learning goal orientation with the cognitive process at the individual psychological level to explore the specific mechanism of subsequent behavioral responses. With this contention in mind, we focused the present study on investigating the affective mechanisms linking learning goal orientation and learning from failure. Among them, the “grief recovery theory” emphasizes the important influence of negative emotions after failure on learning from failure. And its basic logic is “failure events—negative emotions—learning from failure,” emphasizing the important role of negative emotions represented by grief brought by failure in reducing the quality of learning from failure (Shepherd, 2003). Shepherd (2003) and Shepherd et al. (2009, 2014, 2011) based on this theory focused on grief and its recovery mechanism, and proposed several coping orientations for individuals to cope with negative emotions: restoration orientation (a kind of emotion-focused coping orientation), loss orientation (a kind of event-focused coping orientation), and oscillation orientation (alternately use restoration orientation and loss orientation). While goal orientation has an important influence on the internal and external motivations and behavioral responses of individuals (Steele-Johnson et al., 2000). Therefore, according to the grief recovery theory and goal orientation theory, we assume that individuals with learning goal orientation may adopt different types of coping orientations to adapt to the negative emotions brought about by the failure, and the emotional coping behaviors may have a further effect on subsequent learning.

Furthermore, is it possible that some boundary variables will accelerate the effect of the above mechanism? We further anticipate that emotion (e.g., grieving caused by failure) may be crucial to the relationship between cognition (i.e., negative emotion coping orientation) and behavior (i.e., learning from failure) after a failure event has occurred (Dolan, 2002; Phelps, 2006). Grieving will inevitably arise after failure. According to grief recovery theory, the negative emotions represented by grief will affect the breadth and depth of individuals’ information collection and processing, thus reducing the quality of learning from failure. While positive grieving is a form of grieving first proposed by Blau (2006), which describes the positive aspects of grieving, usually manifested in acceptance, exploration, etc. Some previous studies have shown that positive grieving will positively related to learning behavior (Wang et al., 2019), but as a bright aspect of the grieving, few studies use it as a moderator to explore its boundary effect on the learning process. Therefore, exploring whether positive grieving can play a positive role in the relationship between the individual’s coping orientations to negative emotions and learning from failure can help us further understand the mechanism of positive grieving.

**FIGURE 1:**
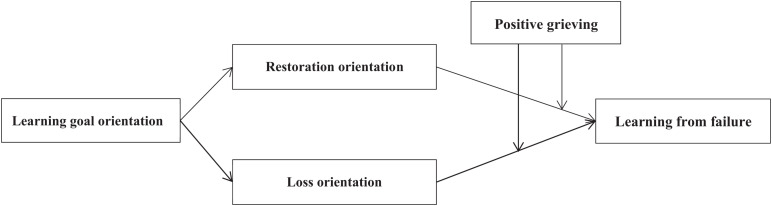
Theoretical model.

In summary, we addressed this issue by testing the conceptual model depicted in Figure 1. First of all, we combined the individual’s deeper-level psychological and cognitive variables to explore the important influence of learning goal orientation on subsequent learning behavior, and further developed the application situation of learning goal orientation mechanism; secondly, by combining with grief recovery theory, we extend the application scope of Shepherd et al. (2011) emotional recovery mechanism, taking the individual’s coping orientation to negative emotions as an mediation variables. Finally, according to the conclusion of Dolan (2002) and Phelps (2006) that emotion plays an important role in the mechanism of behavior, we add a moderation variable, namely positive grieving, which may promote the path of “coping orientation—learning from failure.” Through analyze questionnaire data from high-tech companies in China, in demonstrating the linkages proposed in the model, our results contribute to the literature in several important ways, and also provide practical significance for enterprise management. First, we replicate much of the work reported in Shepherd et al. (2011), among conceptually similar constructs but at the individual’s cognition and behavior level of analysis. Also, to explore the individuals’ deeper level of psychological and behavioral variables (e.g., learning goal orientation and coping orientations), we make learning goal orientation as an antecedent and make coping orientations as a mediation variable to learning from failure. Hence, the current research answers mounting calls for individual-level studies on goal orientation, coping behavior orientations, and learning behavior within an integrated framework. Second, since grieving is inevitable after failure, it is necessary to explore positive grieving (i.e., the positive side of grief) for the occurrence of learning behavior. Our findings may benefit both applied researchers and practitioners, as they reveal a previously unidentified boundary condition regarding the relationship between coping orientations and learning from failure.

## Literature Review and Hypotheses

In fact, previous scholars used learning from failure as an outcome variable to explore the individual-level influencing factors that may affect individual’s learning behaviors. Most of these studies focused on the emotional and cognitive variables generated by individuals after failures events. Regarding the influence of emotional response on learning from failure, Shepherd and Cardon (2009) shows that the individual’s negative emotions after failure will have an impact on learning from failure and following tries. Bohns and Flynn (2013) compared the impact of the emotional response caused by failure on the performance output of employees in the next cycle, and they emphasize that for learning from failure, the emotional response of guilt is more positive and effective than shame. Secondly, the individual’s cognition to failure event will also affect learning from failure. For example, Hao et al. (2018) research shows that critical thinking is beneficial to learning from failure. Boss and Sims (2008) researched that employees’ self-efficacy, emotional regulation and self-leadership can help them recover faster from failure. In addition, failure is not changeless. The number of failures experienced by employees has also become an important influencing factor. For example, Boso et al. (2019) believe that business failure experience will significantly predict learning from failure behavior. Although previous studies have analyzed the influence mechanism of learning from failure from multiple perspectives at the individual level, these variables mostly focus on the emotional or cognitive response after failure, and still lack individual stable psychological factors. Exploring the stable characteristics of individuals can better interpret the cognitive and behavioral processes of ordinary individuals in the face of failure. Specifically, goal orientation theory can well explain the psychological process of the generation of individual’s behavior. Previous scholars have confirmed that different goal orientations lead to different cognitive and behavioral patterns. Dweck’s goal orientation theory represents how personal goals and beliefs create the mental framework from which individuals follow avoidance or approach strategies toward goals, being a distinct construct from both goal setting (e.g., personal choices concerning most attractive goals) and goal striving (e.g., behaviors and thoughts directed toward a specific) (Dweck, 1986; Dweck and Leggett, 1988; Vandewalle and Cummings, 1997). Goal orientation relies on personal beliefs concerning intelligence as either incremental (e.g., learning orientation) or stable (e.g., performing orientation), arguing that these beliefs are responsible for the way individuals apply specific strategies toward the pursuit of goals. performance orientation has shown to possess an avoidance dimension (avoiding failure and to show incompetence) and a performing dimension (choosing to perform easier tasks in order to succeed, showing competence) toward the pursuit of results (Vandewalle et al., 1999; Chen and Mathieu, 2008). Previous research has also confirmed that compared with other goal orientation, learning goal orientation has a variety of positive effects on individual cognition and behavior, such as promoting individual internal motivation (Steele-Johnson et al., 2000), promoting innovation (Hirst et al., 2009), and communicating and cooperating with others (Levy et al., 2004). Therefore, learning goal orientation may also have a positive impact on individual learning from failure behavior. We chose to use learning goal orientation as an antecedent variable that affects individual learning from failure. Exploring this logical relationship can further clarify the stable personal characteristics factors that may promote learning from failure.

Furthermore, combined with the coping-oriented mechanism of individuals coping with negative emotions after failure, we added the mediation variable, that is, the coping orientation of individuals coping with negative emotions. Most of the research on coping orientation is based on the grief recovery theory. Based on this, Shepherd (2003) proposed the coping orientation of individuals to cope with negative emotions. Restoration orientation is a coping strategy that focuses on emotional recovery. Loss orientation is a coping strategy that focuses on event resolution. If an individual alternates using two coping orientations, it is called oscillation orientation. In fact, Shepherd et al. (2011) has proposed that every coping orientation play a moderating role in the relationship between negative emotions and learning from failure, but the conclusions in the article have not been fully confirmed after empirical research. Many scholars have also constructed a theoretical framework based on the coping orientation, and explored the key role of coping orientations in entrepreneurial failure or subsequent entrepreneurial processes. However, few scholars have explored how the coping orientation of emotional response directly affects the learning from failure process. In addition, due to the oscillation orientation integrate the characteristics of restoration orientation and loss orientation, and the generation of oscillation orientation has time continuity, usually manifested as a method of coping with negative emotions on a long-term scale (Shepherd et al., 2011). Therefore, in this article, we only consider the mediating effect of a single loss orientation or restoration orientation, and do not consider the possible mediating effect of oscillation orientation. Therefore, based on the Chinese cultural background, we try to solve this research limitation through empirical research. The contextual factors specific to Chinese culture will cause Chinese employees or managers to show different research results from Western scholars. Many scholars have explored Chinese management culture based on Chinese unique values such as “*mianzi.*” The results show that China’s unique cultural factors will affect individual cognition and behavior patterns from many aspects such as attitude and emotion (Bedford, 2011). Whether this will affect employees’ learning from failure behavior, and how the specific mechanism of this process is still not studied by scholars.

In addition, the concept of grieving was first used in research on commercial failure by Shepherd (2003), who proposed that it is a type of negative emotional response after a failure is experienced. Blau (2006) observes that grieving may be either negative (i.e., denial, anger, and negotiation) or positive (i.e., exploration and acceptance). As a normal emotional response, negative grieving usually appears after a failure event occurs, and often leads to some undesirable consequences such as low performance and low organization citizenship behaviors. There is no doubt that negative grieving will negatively affect the individual’s learning process and behavior. However, as time goes by, there is a transition to positive grieving, which includes an individual’s acceptance and exploration of the event (Blau, 2006); this allows them to make up for the deficiencies caused by negative grieving, which will, in turn, have a positive impact on the individual’s future behavior pattern (Blau, 2007). There are few studies on whether positive grieving has a positive effect or a negative effect on individuals. Some previous studies have shown that positive grieving will positively related to learning behavior (Wang et al., 2019), but as a bright aspect of the grieving, few studies use it as a moderator to explore its effect on the process of “cognition—learning.” When employees are dealing with the impact of negative emotions, can positive grieving have a boundary effect on the learning process? This is very important for employees to learn from failure in a grieving mood. We proposed a different opinion on this question. A coping orientation usually determines the focus of an individual’s use of follow-up resources and strategies, which further influences the occurrence of subsequent behavior patterns (Shepherd et al., 2011), which in turn will be affected by individual emotions. Because positive grieving has been shown to have a positive effect on individuals, we try to further explore its mechanism on learning from failure (Blau, 2007). Therefore, we assume that positive grieving can moderate the process mechanism of the relationship between coping orientation to learning from failure. Based on the above, we have constructed a theoretical model with coping orientation and positive grieving as a mediator and boundary variable, we will systematically explain this model below.

### Learning Goal Orientation and Learning From Failure

According to the goal orientation theory, an individual’s learning goal orientation will have a positive impact on that individual’s behavior (Cury et al., 2006). Individuals with a learning goal orientation mainly focus on behavioral processes related to learning and tasks (Zweig and Webster, 2004). Therefore, we believe that a learning goal orientation will promote individual behavior that helps them learn more from failure.

Individuals with a high learning goal orientation believe that abilities can be improved through learning (Dweck, 1999). With persistence and hard work, anyone can solve and overcome difficulties, develop their ability, and achieve better success in future tasks (Dweck, 1999). Rather than worrying about the adverse effects of failure, they are more interested in improving their ability (Levy et al., 2004). Therefore, people with a high learning goals orientation are more likely to persist in learning after failure events, continue to work hard, summarize their experience in order to further develop their ability, and achieve future improvements.

LGO will affect peoples’ perceptions of event feedback (Nisan, 1972). Individuals with a high LGO view feedback as useful because it provides information about events. Understanding this information and learning from it allows more effective completion of future tasks (Dahling and Ruppel, 2016). For individual with a high LGO, negative feedback is seen as a challenge and provides motivating information. If we can learn from it, we can make ourselves better (Dweck, 1986). When individuals with a high LGO receive negative feedback, they continue to work hard to find solutions (Dweck and Leggett, 1988). Overall, individuals with a high LGO regard failure as an opportunity to develop themselves, and when encountering failure events, they try to learn from them. Therefore, we propose the following hypotheses:

*Hypothesis 1: Individual employees who have a high LGO will learn more from failure than individual employees who have a low LGO.*

### The Mediation of Restoration Orientation

Goal orientation theory says that an individual’s goal orientation will stimulate motivation, which in turn will influence behavior. Therefore, we propose that, as a stable behavioral orientation, the individual’s learning goal orientation will affect the individual’s coping response (e.g., restoration or loss orientation) after failure occurs.

A restoration orientation refers to “the suppression of feelings of loss and proactiveness toward secondary sources of stress that arise from a loss” (Shepherd et al., 2011). Individuals with a strong learning goal orientation pay more attention to the development of abilities (Dweck, 1986). They are willing to try to achieve challenging goals, possess a strong internal motivation and autonomy, and actively look for opportunities for learning and creation in a future work environment (Van Yperen, 2003). Therefore, we assume that a high learning goal orientation will lead to a restoration orientation.

A restoration orientation focuses on recovering from negative events by diverting attention away from failure events and toward other goals (Shepherd et al., 2011). Employees tend to avoid major stressors like project failures and deal with secondary stressors by “cleaning up negative consequences caused by project failures” (Stroebe and Schut, 1999). Seijts and Latham (2006) found that individuals with a high learning goal orientation collect additional information to obtain to improve their capabilities. In addition to the negative impact of project failure, it also brings challenging task requirements and follow-up work tasks (Shepherd et al., 2011), which provides employees with follow-up learning goals and tasks. Thus, the derivative problems caused by failure become an important source of learning, and the experiences and lessons learned from them can become an important source of individual knowledge and skill development (Stroebe and Schut, 1999). Therefore, individuals with a high learning goal orientation may divert their attention from the failure event, and actively engage in the handling of external events (e.g., follow-up challenging tasks and works), that is, take a restoration orientation.

Additionally, individuals with a high learning goal orientation are sensitive to information that may help them (Dahling and Ruppel, 2016). They usually hold the view that “ability can be changed,” thinking that ability can be increased through continuous learning from various events related to failure, so they often have self-confidence in their ability (Dahling and Ruppel, 2016). They are eager to enhance their internal motivation to learn through a series of challenging events brought on by failures, and then improve their abilities (Dweck and Leggett, 1988). For the derivative problems caused by failure, they will also be considered as a way of learning to strengthen the learning of experience in failure to improve personal ability (Dahling and Ruppel, 2016), which will prompt individuals to turn to solve the derivative problems (that is, external events), and continue to pay attention to the “secondary stressors” brought on by failed events. Therefore, we assume that:

*Hypothesis 2a: There is a positive relationship between a high learning goal orientation and a restoration orientation.*

Eastern culture usually pays attention to “mianzi” (also called “Face” or “Lian”), which is a unique cultural characteristic (Bedford and Hwang, 2003), often be interpreted as both the showing of respect (“giving face”) and ensuring that you do not offend people (causing them to “lose face”). It is a positive public image that a person conveys to others (Ting-Toomey, 1994). Factors such as external stimulus events will increase the individual’s motivation to maintain “mianzi,” and then make corresponding behavioral responses (Hwang, 1987). When facing negative events (i.e., failure events), individuals immerse themselves in a series of negative effects will influence their maintenance of “mianzi,” and increase their fear that they will be looked down upon by others (Jiang, 2006). Implementing a restoration orientation can help people divert attention away from negative events, buffer the negative effects caused by the failure, and thereby provide employees with new information about failures and a new perspective on overcoming failures (Shepherd et al., 2011).

As Yamakawa and Cardon (2015) note, individuals usually produce learning behavior through multiple links such as scanning (i.e., selectively paying attention to, and collecting important information about, failure) and interpretation (processing the scanned information for easy understanding). The two aspects of a restoration orientation—“proactiveness restoration” (i.e., proactively solving the derivative problems caused by a failure) and “avoidance restoration” (i.e., diverting attention away from the failure) are intertwined (Shepherd et al., 2011). When proactively solving a series of problems that derive from a failure, individuals can obtain information about the failure, which is conducive to information scanning (Cope, 2011). When using avoidance restoration, individuals will be free from the negative effects of the failure (such as negative emotions), will enhance the information processing ability, and promote the interpretation of the failure event (Shepherd et al., 2011). Every level can shift the attention to events other than failure, pay more attention to a series of challenges brought by failure, which will reduce the negative emotions caused by loss of “mianzi.” By scanning and interpreting failures and follow-up events, individuals can enhance their ability to construct the meaning of a failure, which helps individuals better understand failures and learn from them (Shepherd and Cardon, 2009). Therefore, we assume that:

*Hypothesis 2b: There is a positive relationship between a restoration orientation and learning from failure.*

H2a predicted the positive relationship between learning goal orientation and restoration orientation, and H2b predicted the positive relationship between restoration orientation and learning from failure. In conclusion, we also assume that individuals with a high learning goal orientation will trigger a restoration orientation, which will reduce the negative effects of the failure and stimulate positive behavior (i.e., learning from failure). Thus, we assume that a restoration orientation is a mediator in the relationship between learning goal orientation and learning from failure:

*Hypothesis 2c: A restoration orientation mediates the relationship between a learning goal orientation and learning from failure.*

### The Mediation Effect of a Loss Orientation

A loss orientation refers to “working through and processing aspects of a loss” (Shepherd et al., 2011). Some individuals with loss orientation regard failure as an important learning resource (Shepherd et al., 2011). Learning knowledge, skills, and experience from failures will help individuals improve their abilities to deal with similar tasks (Dweck, 1986). We assume that there is a close relationship between a learning goal orientation and loss orientation.

People with a high learning goal orientation value the plasticity of ability, and they believe that they can change the direction of events and improve their ability through their hard work (Dweck, 1999). So they pay more attention to the failure and tend to invest more effort in handling failure events, such as exploring the cause of the failure and suppressing the negative emotions caused by the failure (Dweck and Leggett, 1988). When individuals with a high learning goal orientation deal with work issues, they often use a task involvement strategy, which means actively participating in issue processing in order to meet the needs of the work role. When they experience failure, they will immerse themselves in the event (VandeWalle et al., 2001), that is, adopt a loss orientation.

Failure events usually bring on negative emotions, and these negative emotions will make individuals avoid future failures (Iyer et al., 2007). Individuals with a high learning goal orientation regard negative feedback as an opportunity to make progress in their life. They face the negative feedback with confidence, ignore the negative emotions brought on by failure, and weaken the impact of negative emotions by self-adjusting (Dweck and Leggett, 1988). They investigate the cause of failure and try to determine what went wrong. In doing so, they are sensitive to information that can help them to develop (Dahling and Ruppel, 2016). Although a failure event indicates that the individual’s ability is lacking in some way, it also makes the individual aware of valuable information contained in the failure event (VandeWalle et al., 2001). Rather than regard failure as a blow, individuals are more likely to regard failure as an opportunity to learn new skills. They will explore the reason of failure, search and summarize relevant information to achieve personal development (Dahling and Ruppel, 2016), thus strengthening their loss orientation. Therefore, we assume that:

*Hypothesis 3a: A high learning goal orientation has a positive relationship with a loss orientation.*

Shepherd et al. (2011) found that a loss orientation includes two dimensions: a “self-dimension” (which focuses on the failure process and investigates the reason for the failure), and an “others-dimension” (which involves communicating with the outside world to discover the reason for the failure). In the “self-dimension,” individuals who adopt a loss orientation after failure will pay more attention to the failure and its reasons. Although they will also face negative emotions such as sadness and inferiority caused by the failure, those who adopt a loss orientation will not mindlessly engage in negative thinking from which they cannot extricate themselves (Shepherd et al., 2011). They are able to quickly break the relationship between a bad mood and failures and make the transition to a stable mood as they reflect on their failure (Shepherd et al., 2011). By exploring the reasons for a failure, individuals can understand failure deeply (Corbett et al., 2007), have a better understanding in the errors or limitations in the failed project (Birtchnell, 2001), and make an objective attribution (Baron, 2000).

In the “others-dimension” of loss orientation, individuals who adopt a loss orientation after failure tend to talk about their feelings regarding the project failure to their friends and family, and find out the reasons for the failure by asking others for their opinions (Shepherd, 2003). This helps them explore and accept the reasons for the failure, increases their confidence, and prepares them to make corresponding adjustments to improve their present situation (Rybowiak et al., 1999). All of this helps them learn from failure.

Investigating the reasons of failure and its solutions will make individuals aware of the potential value of failure and help them to integrate relevant and useful information. Individuals who adopt a loss orientation will tend to regard failure as an opportunity to improve skills and develop themselves (Tjosvold et al., 2004). Such an orientation will help the individual have a positive cognitive assessment of failure, and encourage them to learn from failure. From the perspective of eastern culture, whether people take measures of correct attribution or cognitive assessment, they can maintain their positive images or social status, which is an effective way for them to pursue subsequent learning. Therefore, we assume that:

*Hypothesis 3b: A loss orientation has a positive relationship with learning from failure.*

H3a predicted the positive relationship between learning goal orientation and loss orientation, and H3b predicted the positive relationship between loss orientation and learning from failure. Together, these hypotheses specify a model in which a learning goal orientation indirectly increase learning from failure by contributing to a loss orientation. We assume that individuals with a high learning goal orientation will devote themselves to the summary of failure events and further participate in the follow-up treatment of failure events will help them become immersive, further explore the experiences and lessons learned from failures, promote them to face failures and learn from failures. Therefore, loss orientation is another mediator in the relationship between learning goal orientation and learning from failure:

*Hypothesis 3c: A loss orientation mediates the relationship between a learning goal orientation and learning from failure.*

### The Moderation Effect of Positive Grieving

Individuals will experience some negative emotions (e.g., guilt, anger, and shame) after a failure (Carver and Scheier, 1990), and these emotions can strengthen or weaken learning behavior (Zhao and Olivera, 2006; Shepherd and Cardon, 2009). Dolan (2002) and Phelps (2006) argue that emotion will play an important role in the learning process, and will have an impact on cognition and behavior after a failure occurs. Grief is a negative emotional response (Shepherd, 2003), but positive grieving is the bright side of grief. According to Blau (2007), positive grieving manifests itself in two ways: exploration (i.e., for hopeful opportunities and new possibilities), and acceptance (i.e., accepting the fact of failure). Individuals with positive grieving will accept the facts of failure, helping them shift the attention away from failure events (Ellard et al., 2017). Individuals with a high restoration orientation are good at dealing with external or derivative information regarding failure, and pay more attention to other goals (Shepherd et al., 2011). Therefore, they can further enhance their motivation to learn from outside the failure event. In addition, individuals with high positive grieving are not afraid of failure, and tend to easily escape from its negative effects (Blau, 2007). From a resource perspective, they will have more cognitive resources to deal with the external or derivative information (Shepherd, 2003). Individuals with positive grieving will show more constructive behaviors, such as communicating with colleagues to conclude the experience and lessons from failure, which will help them invest in the next project task earlier (Blau, 2007). These benefits will help individuals with a restoration orientation to enhance their learning behaviors.

By contrast, individuals with a loss orientation usually focus more on information about the failure event. They tend to explore the reasons for the failure, and continuously search for information about the failure. From two perspectives of positive grieving, in exploration, individuals with high positive grieving usually have a bright view of the future, and pay more attention to future tasks and work opportunities (Blau, 2007). Limited cognition resources will not be used to obsess about the failure, and less attention will be paid to the failure. This will weaken the motivation of such individuals to learn from the failure, which in turn will weaken the relationship between loss orientation and learning from failure. With respect to acceptance, accepting the fact of failure will help individuals to shift their attention from concentrating on the negative events to reflecting on the significance of the event (Ellard et al., 2017). With an acceptance, individuals will reduce their excessive attention on the failure, and they will come to regard failures as “normal events,” This will weaken the motivation of learning from failure events. Therefore, we propose the following hypotheses:

*Hypothesis 4a: Positive grieving positively moderates the relationship between a restoration orientation and learning from failure, i.e., the relationship will be stronger when positive grieving is higher, rather than when it is lower.*

*Hypothesis 4b: Positive grieving negatively moderates the relationship between a loss orientation and learning from failure, i.e., the relationship will be weaker when positive grieving is higher, rather than when it is lower.*

## Materials and Methods

### Participants and Procedure

As the R&D teams of high-tech companies are more likely to encounter setbacks and failures in the R&D process, most technology-based employees may have experienced failures, and the sample is more representative than other industries. Therefore, we focus on high-tech firms in China as our research participants. We define a firm as “high-tech” if 60% or more of its annual sales revenues come from high-tech products and services, and if 10% or more of its employees have engaged in R&D in the past year. According to this standard, we randomly selected 400 high-tech enterprises from the list of Beijing high-tech enterprises provided by the Beijing Municipal Science and Technology Commission, and we invited them to participate in the research during an introductory telephone conversation. The participants are members of the R&D teams in these companies. These teams are required to have participated in project development in the past 3 years and have had the experience of project failure. During the phone call, we emphasized the purpose of the research and the confidentiality of data collection. We then asked the CEO to write an endorsement to encourage employees to participate in a questionnaire survey, and we promised to give the final research results to the companies’ leaders.

The distribution and recovery of the questionnaires was accomplished using the following steps. First, the firms that participated in the study selected a coordinator (usually a human resource manager) who provided our research assistant with a list of research teams (usually R&D teams). With the help of the coordinator, the research assistant distributed questionnaires to the staff before the weekly (or monthly) regular meeting of the team. To ensure everyone’s participation, the research assistant obtained contact information from the coordinator for any members who missed meetings. An envelope was left for these absentees to fill out and return to the research assistant. In order to improve the recovery rate of the research questionnaire, we also distributed small gifts and the endorsement of the CEO. After answering the questionnaire, all participants signed a confidentiality agreement to ensure that the questionnaire was not used for other purposes.

The final sample included 22 companies in Beijing area technology industry (750 responses in total). All team leaders and members provided completed questionnaires. The average team size, including the team leader, was 5.43, and ranged from 3 to 10 (*SD* = 1.60). The mean respondent age was 31.67 years (age range was 20–56 years, *SD* = 5.525), with 577 men (79%) and 173 women (21%). About 51.2% of the respondents had bachelor’s degrees and 38.5% had a master’s or doctor’s degrees, and the remaining samples are all college degrees.

### Measures

In this study, we defined project failure depending on the results of research projects. Following previous studies, we defined project failure as ‘the termination of an initiative to create organizational value that has fallen short of its goals’ (McGrath, 1999; Hoang and Rothaermel, 2005), and we gave this definition in the introduction section of our questionnaires. We first arrange and organize the original scales, and then use the back-translation method (Brislin, 1970) to ensure that there will be no translation errors. All coefficient alpha is Cronbach’s alphas. All scales are scored by using the Likert-6 scale.

#### Learning Goal Orientation

We used the five-item scale developed by Vandewalle and Cummings (1997) to measure learning goal orientation. It asks employees to explain how they learn from a project failure. Sample items include “I am willing to choose those challenging tasks,” and “I often seek opportunities to develop new skills and learn new knowledge.” Response options ranged from 1 (strongly disagree) to 6 (strongly agree). The coefficient alpha of the scale was 0.852.

#### Restoration Orientation

We used the six-item scale developed by Shepherd et al. (2011) to measure restoration orientation. It asks employees to explain to what extent they agree with each behavior statement after a failure. Sample items include “I intentionally divert my attention, not thinking about the problem of the project failure” and “After the project fails, I try to sort out my thoughts.” Response options range from 1 (strongly disagree) to 6 (strongly agree). The coefficient alpha of the scale was 0.636.

#### Loss Orientation

We used the six-item scale developed by Shepherd et al. (2011) to measure loss orientation. It asks employees the extent to which they agree with the behavior statement after a failure. Sample items include “I worked with my colleagues to find the cause of the failure” and “I worked hard to overcome the negative emotions associated with the failure of the project.” Response options range from 1 (strongly disagree) to 6 (strongly agree). The coefficient alpha of the scale was 0.696.

#### Positive Grieving

We used the six-item scale developed by Blau (2007) to measure positive grieving. It asks employees to state their personal acceptance of failed projects. Sample items include “I accept the reality of project failure” and “I am willing to explore other possibilities from failed projects.” Response options range from 1 (strongly disagree) to 6 (strongly agree). The coefficient alpha of the scale was 0.833.

#### Learning From Failure

We used the eight-item scale developed by Shepherd et al. (2011) to measure learning from failure. Employees are asked to explain the degree of change in their own behavior after a failure, including both personal and project dimensions. Sample items include “I have learned to execute the project plan better” and “I have improved my ability to make more contributions to new projects.” Response options ranged from 1 (strongly disagree) to 6 (strongly agree). The coefficient alpha of the scale was 0.907.

#### Control Variables

Beyond the demographic variables (i.e., gender, age, education level, and tenure in the firm and on a project), we also controlled for the critical factors in project failure and the parallel variables of the variables in the following model: performance-approach goal orientation, performance-avoidance goal orientation, oscillation orientation, and negative grieving. These are described below.

##### Critical factors in project failure

We used the two-item scale adapted by Dilts and Pence (2006) (According to the results of exploratory factor analysis (EFA), the original 13 items are divided into two items for internal and external factors) to measure the critical factors in project failure. The instrument asks employees to explain why they think the project failed. Sample items include “change in the importance of the entire project in the organization,” and “changes in user needs.” Response options ranged from 1 (strongly disagree) to 6 (strongly agree). The coefficient alpha of the scale was 0.656.

##### Performance-approach goal orientation

We used the four-item scale developed by Vandewalle and Cummings (1997) to measure performance-approach goal orientation. It asks employees to explain their personal strategy for improving their performance after a failure has occurred. Sample items include “I tried to find a way to prove my ability to colleagues” and “I am willing to do projects that can prove my ability to others.” Response options ranged from 1 (strongly disagree) to 6 (strongly agree). The coefficient alpha of the scale was 0.789.

##### Performance-avoidance goal orientation

We used the four-item scale adapted by Vandewalle and Cummings (1997) to measure performance-avoidance goal orientation. Because the reliability of the original scale in our research is not enough, we deleted one of the items to improve the reliability. The instrument asks employees to state their personal strategy for avoiding the possibility of failure. Sample items include “I am not willing to take on a task that may show my lack of ability” and “When performing a task, I just try to avoid showing incompetence.” Response options ranged from 1 (strongly disagree) to 6 (strongly agree). The coefficient alpha of the scale was 0.820.

##### Oscillation orientation

An oscillation orientation involves moving between using the restoration orientation and using the loss orientation. We used the three-item scale developed by Shepherd et al. (2011) to measure oscillation orientation. It asks employees the extent to which they agree with their statement of behavior after a failure has occurred. Sample items include “After giving my emotions a rest, I confront my negative feelings arising from the project’s failure” and “After thinking about the failure for a period of time, I try not to think about it as much as possible.” Responses options ranged from 1 (strongly disagree) to 6 (strongly agree). The coefficient alpha of the scale was 0.580. Because the alpha of this variable is low, we removed this variable and did another data test. The results showed that the existence of this variable did not have much impact on the data test results (the data results are shown in the Appendix).

##### Negative grieving

We used the six-item scale adapted by Blau (2007) to measure negative grieving. Because the reliability of the original scale was low, we deleted one of the items to improve the reliability. The instrument asks employees to state their acceptance of failed projects. Sample items include “I can’t believe this will happen to me” and “I’m depressed for the failure of the project.” Responses options ranged from 1 (strongly disagree) to 6 (strongly agree). The coefficient alpha of the scale was 0.864.

## Results

In this study, we used Amos 24.0, SPSS 25.0, and Stata 12.0 for data analysis and hypothesis testing. We analyzed the validity of the measurement model, the basic distribution of data, the correlation between variables, and the reliability of the scale. We also do multiple linear regression analysis to test our hypotheses. We use Harman’s single factor analysis to test whether the data has serious common method bias. The results show that the interpretation rate of the first common factor is less than 40% (18.66%, so our data does not have serious common method bias).

### Confirmatory Factor Analysis

We first conducted a confirmatory factor analysis (CFA). As shown in Table 1, our theoretical model (10-factor model) fits better (CMIN/df = 2.470, CFI = 0.909, RMSEA = 0.044) than other models, indicating the construct distinctiveness of our measurements.

**TABLE 1:** Comparison of measurement model.

Model	CMIN	DF	CMIN/DF	IFI	TLI	CFI	RMSEA
Theoretical model (Ten-Factor Model) (LGO, AvoGO, AppGO, RO, LO, OO, PGri, NGri, LFF, Factor)	2682.009	1086	2.470	0.910	0.893	0.909	0.044
Eight-Factor Model (GO, RO, LO, OO, PGri, NGri, LFF, Factor)	3682.041	1103	3.338	0.854	0.83	0.853	0.056
Six-Factor Model (GO, Orientation, PGri, NGri, LFF, Factor)	3781.353	1116	3.388	0.849	0.826	0.848	0.056
Five-Factor Model (GO, Orientation, Gri, LFF, Factor)	4538.198	1121	4.048	0.807	0.779	0.805	0.064
Four-Factor Model (GO + Orientation, Gri, LFF, Factor)	5250.728	1125	4.667	0.767	0.734	0.765	0.070
Three-Factor Model (GO + Orientation + Gri, LFF, Factor)	5163.035	1128	4.577	0.772	0.74	0.770	0.069
Two-Factor Model (GO + Orientation + Gri + LFF, Factor)	5424.609	1130	4.801	0.757	0.724	0.755	0.071
One-Factor Model (GO + Orientation + Gri + LFF + Factor)	5625.266	1131	4.974	0.746	0.711	0.744	0.073

*LGO refers to learning goal orientation, AvoGO refers to performance-approach goal orientation, APPGO refers to performance-avoidance goal orientation, RO refers to restoration orientation, LO refers to loss orientation, OO refers to oscillation orientation, PGri refers to positive grieving, NGri refers to negative grieving, Gri refers to grieving, LFF refers to learning from failure, Factor refers to critical factors in project failure.*

### Descriptive Statistics

Table 2 shows the descriptive statistics and the correlations among the variables. As shown in the table, learning goal orientation is significantly related to restoration orientation (*r* = 0.145, *p* < 0.01), loss orientation (*r* = 0.174, *p* < 0.01), and learning from failure (*r* = 0.368, *p* < 0.01). Restoration orientation is significantly related to learning from failure (*r* = 0.390, *p* < 0.01), and loss orientation is significantly related to learning from failure (*r* = 0.439, *p* < 0.01). The data results indicate that there may be a close relationship between learning goal orientation, coping orientation, and learning from failure.

**TABLE 2:** Means, standard deviations, reliability, and correlations among study variables.

	Mean	*SD*	1	2	3	4	5	6	7	8	9	10	11	12	13	14	15	16	17
(1) Gender	1.23	0.421																	
(2) Age	31.67	5.525	–0.050																
(3) Education	4.36	0.677	–0.007																
(4) Years in the firm	1.76	0.735	–0.014	0.687**	0.187**														
(5) Years in the team	1.46	0.691	–0.043	0.496**	0.218**	0.768**													
(6) Position	4.31	2.691	.037	−0.079*	−0.120**	−0.140**	−0.180**												
(7) Type of R&D work	2.97	0.756	.112	–0.032	−0.124**	–0.064	−0.101**	–0.008											
(8) Reason for project failure	2.98	0.982	–0.028	–0.038	−0.098**	–0.066	–0.047	0.034	0.003	(0.656)									
(9) Learning goal orientation	4.73	0.698	–0.033	–0.062	–0.07	−0.087*	–0.058	–0.045	0.06	0.011	(0.852)								
(10) Performance-approach goal orientation	4.60	0.718	–0.036	0.021	–0.041	0.039	0.033	–0.004	0.049	–0.002	0.574**	(0.789)							
(11) Performance-avoidance goal orientation	3.07	0.941	–0.066	0.076*	–0.02	0.071	0.04	–0.012	0.011	0.133**	−0.134**	0.101**	(0.820)						
(12) Restoration orientation	3.88	0.655	0.033	0	–0.06	–0.053	–0.05	–0.005	0.056	0.104**	0.145**	0.123**	0.157**	(0.636)					
(13) Loss orientation	3.92	0.724	0.06	0.01	−0.136**	–0.037	–0.058	0.028	0.034	0.066	0.174**	0.162**	0.002	0.470**	(0.696)				
(14) Oscillation Orientation	4.02	0.852	.072*	0.001	–0.056	–0.013	0.014	–0.045	0.075*	0.02	0.146**	0.168**	0.03	0.651**	0.403**	(0.580)			
(15) Positive Grieving	4.35	0.839	0.06	0.004	–0.057	–0.046	–0.066	0.004	0.134**	0.032	0.304**	0.163**	−0.179**	0.375**	0.369**	0.414**	(0.833)		
16 Negative Grieving	2.90	0.877	−0.075*	0.028	−0.095**	0.028	–0.025	0.032	0.025	0.261**	−0.113**	–0.027	0.217**	0.253**	0.219**	0.151**	0.015	(0.864)	
(17) Learning from failure	4.58	0.830	0.056	–0.024	–0.031	–0.032	–0.037	–0.021	0.091*	–0.063	0.368**	0.249**	−0.116**	0.390**	0.439**	0.425**	0.542**	−0.103**	(0.907)

*p < 0.05; **p < 0.01.

### Hypothesis Testing

Since the research participants come from different companies, in order to test whether the company environment will affect the research results, we compared the results of the hierarchical regression in SPSS 25.0 and the results of the hierarchical regression after using the cluster statement to control the company variables in Stata 12.0. We found that there is a slight difference between the two regression results (results of Stata are shown in the Appendix), but the effect of learning goal orientation on learning from failure behavior is similar in different companies’ employees. We used SPSS 25.0 for data processing to test our hypotheses, and the result of the hierarchical regression is shown in Table 3.

**TABLE 3:** Hierarchical regression analysis for models.

	Restoration orientation		Loss orientation		Learning from failure						
			
Variables	Model 1.1	Model 1.2	Model 2.1	Model 2.2	Model 3.1	Model 3.2	Model 3.3	Model 3.4	Model 3.5	Model 3.6	Model 3.7
Gender	0.083	0.129	0.166	0.189*	0.111	0.138	0.031	–0.015	–0.017	0.001	0.015
Age	0.015	0.013	0.014	0.014	0.000	0.000	–0.007	–0.010	–0.009	–0.007	–0.008
Education	–0.070	–0.053	–0.203	−0.188**	–0.025	0.000	0.073	0.052	0.050	0.052	0.057
Years in the firm	–0.130	–0.124	–0.095	–0.079	–0.044	0.002	0.061	0.086	0.083	0.074	0.077
Years in the team	–0.005	–0.006	–0.009	–0.016	–0.006	–0.019	–0.033	–0.031	–0.031	–0.037	–0.040
Position	–0.010	–0.007	–0.003	–0.001	–0.011	–0.005	0.001	0.000	0.000	0.000	0.000
Type of R&D work	0.050	0.036	0.009	–0.005	0.112*	0.088	0.072	0.039	0.037	0.027	0.029
Reason for project failure	0.084*	0.063	0.080*	0.082*	–0.058	–0.044	−0.072*	–0.045	–0.046	–0.044	–0.043
Learning goal orientation		0.150**		0.132**		0.322***	0.254***	0.164***	0.165***	0.163***	0.159***
Performance-approach goal orientation		0.025		0.084		0.072	0.022	0.025	0.027	0.033	0.032
Performance-avoidance goal orientation		0.168***		–0.001		–0.069	−0.097**	–0.018	–0.019	–0.028	–0.030
Restoration orientation							0.126**	0.108**	0.105**	0.094*	0.098*
Loss orientation							0.253***	0.232***	0.230***	0.230***	0.233***
Oscillation orientation							0.206***	0.138***	0.136***	0.129**	0.131**
Positive grieving								0.306***	0.300***	0.289***	0.300***
Negative grieving								−0.168***	−0.169***	−0.164***	−0.158***
Restoration orientation × Positive grieving									–0.016		0.052*
Loss orientation × Positive grieving										−0.080***	−0.115***
*R*^2^	0.008	0.052	0.025	0.058	0.005	0.145	0.355	0.446	0.455	0.455	0.457
△*R*^2^		0.044		0.033		0.14	0.21	0.091	0.009	0	0.002
*F*	1.72	4.622	3.308	5.097	1.484	12.228	29.632	37.613	35.415	36.808	35.127
*P*	0.090	0.000	0.001	0.000	0.159	0.000	0.000	0.000	0.000	0.000	0.000

*p < 0.05; **p < 0.01; ***p < 0.001.

Hypothesis 1 proposed a positive relationship between a learning goal orientation and learning from failure. As shown in Table 3, the coefficient between learning goal orientation and learning from failure is significant (*b* = 0.322, *p* < .001, Model 3.2); this supports Hypothesis 1. Hypothesis 2a posited that learning goal orientation is positively associated with restoration orientation. As shown in Table 3, the relationship between learning goal orientation and restoration orientation is significant (*b* = 0.150, *p* < 0.01, Model 1.2). Hypothesis 2b posited that restoration orientation is positively associated with learning from failure. In Table 3, the relationship between restoration orientation and learning from failure is significant (*b* = 0.126, *p* < 0.01, Model 3.3). These results provide support for both hypotheses 2a and 2b. Hypothesis 3a proposed that learning goal orientation is positively associated with loss orientation. As shown in Table 3, the relationship between learning goal orientation and loss orientation is significant (*b* = 0.132, *p* < 0.01, Model 2.2). Hypothesis 3b proposed that loss orientation is positively associated with learning from failure. In Table 3, the relationship between loss orientation and learning from failure is significant (*b* = 0.253, *p* < 0.001, Model 3.3). These results provide support for hypotheses 3a and 3b. Hypothesis 2c and 3c assumed a mediating role of restoration orientation and loss orientation. We use the Macro program Process developed by Hayes for Bootstrap test to further examine the mediating role of restoration orientation and loss orientation. Our results show that the indirect effect of restoration orientation is.0173 (95% CI [0.0042–0.0361]) and the indirect effect of loss orientation is.0353 (95% CI [0.0102–0.0603]); these results provide support for hypothesis 2c and 3c.

Hypotheses 4a and 4b proposed a moderating role of positive grieving. As shown in Table 3, when the interactive items (positive grieving × restoration orientation and positive grieving × loss orientation) are entered at the same time, the coefficients of the two are both significant (*b*_*RO*_ = 0.052, *p*_*RO*_ < 0.05, *b*_*LO*_ = –0.115, *p*_*LO*_ < 0.001, Model 3.7). When the interaction items are entered separately, the coefficient of positive grieving × loss orientation is also significant (*b* = –0.08, *p* < 0.001, Model 3.6), but the coefficient of positive grieving × restoration orientation is not significant (*b* = –0.016, *p* > 0.05, Model 3.5). Thus, hypothesis 4b is supported, but hypothesis 4a is rejected. We also test the moderated mediation effect by Process. The results show that the moderating effect of positive grieving on the relationship between restoration orientation and learning from failure is not supported (index^1^ = 0.006, 95% CI [–0.0005–0.0145]). Instead, the moderating effect of positive grieving on the relationship between loss orientation and learning from failure is supported (index = –0.0143, 95% CI [–0.029 to –0.0034]).

**FIGURE 2:**
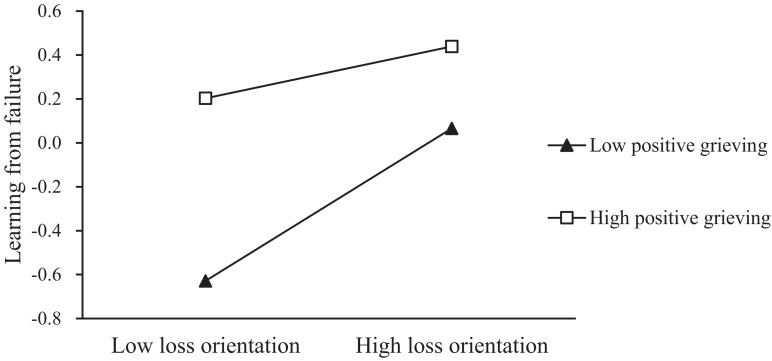
The moderating role of Positive Grieving between Loss Orientation and Learning from Failure.

In order to better interpret the moderating role of positive grieving between loss orientation and learning from failure, following Cohen and Cohen (1983), we define high and low positive grieving as plus and minus one standard deviation from the mean. As shown in Figure 2, for individuals with a higher level (1 SD above the mean) of positive grieving, their loss orientation will take more learning from failure behaviors (*b* = 0.349, *p* < 0.05) than those with a lower level of positive grieving (*b* = 0.119, *p* < 0.05).

## Analysis and Discussion

In this study, we analyzed how a learning goal orientation promotes learning among team members after a project failure occurs. Our results show that both a restoration coping orientation and a loss coping orientation mediate the relationship between a learning goal orientation and learning from failure. Positive grieving negatively moderates the relationship between a loss orientation and learning from failure, but a hypothesis that positive grieving moderates the relationship between a restoration orientation and learning from failure is not supported.

### Theoretical Contribution

The theoretical contributions of the current study are threefold. First, our study enriches the research on the antecedent variables of learning from failure. Past research has focused mostly on the cognitive reactions after failure (Zhao and Olivera, 2006; Shepherd and Cardon, 2009), little is known about the effects of a stable mindset on failure (i.e., goal orientation). This study focuses on the impact of individual behavior orientation on learning from failure. The results of the study validate the role of individual learning goal orientation in promoting learning after a failure. According to the goal orientation theory, an individual’s behavioral orientation will directly or indirectly affect the individual’s behavior (Dweck and Leggett, 1988). However, previous studies tend to pay more attention to variables at the surface-level (such as team atmosphere and individual personality), and there is little research that is focused on individuals’ deep-level attributes such as behavioral orientation. Therefore, this research provides a new research direction.

Second, combined with the theory of grief recovery theory, we extend the application environment of the theory. Shepherd et al. (2011) used grief recovery as a boundary variable to explore the moderating role of emotional coping orientation in the recovery of negative emotions, and believed that loss orientation can promote learning from failure while restoration orientation cannot promote learning. However, after empirical testing, the results have not been fully confirmed. Based on the Chinese cultural background, we propose and test that loss orientation and restoration orientation both have a positive mediating effect in the relationship between learning goal orientation and learning from failure. This expands the context of the grief recovery theory, and subsequent scholars can carry out qualitative research such as case analysis to further confirm its reliability.

Third, an emotional response after failure will influence individuals’ behavior responses (Dolan, 2002; Phelps, 2006). We explored the important role of positive grieving generated by individuals interacting with projects. Regarding to the boundary factors that affect the learning from failure process, previous scholars usually limit the moderating variables to the individual’s stable emotions (e.g., shame, shame, etc.), the organization’s management style (e.g., error management culture), and individual characteristics (e.g., resilience), there is little research on the role of failure-induced transitional emotions (i.e., positive grieving) in individual learning process (Zhao, 2011; Fang He et al., 2018). As a normal emotion after negative events occur, previous research shows that positive grieving may have an impact on learning behaviors (Wang et al., 2019). However, few scholars use it as a moderating variable to study its influence on the mechanism of individual behavior and its antecedent variables. We further expand the research field of positive grieving. After data testing, the negative moderating role on the relationship between loss orientation and learning from failure has been supported. This is very different from the research conclusions of previous scholars. Most of the previous scholars have confirmed that positive grieving has a positive effect (e.g., learning from failure) (Wang et al., 2019). Our research confirms that positive grieving may also have a negative effect, which provides a theoretical and practical basis for follow-up scholars to further explore. However, the moderating role on the relationship between restoration orientation and learning from failure hasn’t been supported. We contend that our cross-section design contributes to this result. In fact, the benefits of a restoration orientation require time to manifest (Shepherd et al., 2011). Such an orientation cannot buffer the negative effects of failure in the short-term in the way that a loss orientation can. Therefore, our findings reveal the potential difference between loss and restoration orientation, which bears further empirical examination in the future.

### Practical Contributions

In terms of management practice, this study suggestions the following recommendations. First, the research results show that individuals with a higher learning goal orientation are more concerned about the development of their abilities and are willing to work hard to improve them (VandeWalle et al., 2001) so that they can learn better from failure. Therefore, team leaders and managers can introduce incentives to encourage staff members to improve themselves. Such a system will increase performance and rewards, motivate a learning goal orientation, and help individuals to learn from failure.

Our research also shows that both restoration and loss orientations can promote learning behavior. Managers should guide and encourage employees to take appropriate countermeasures after a failure occurs. Employees should consciously adopt appropriate treatment methods to maximize the value of their experience and skill learning that failure events provide.

Finally, managers need to realize that positive grieving affects the relationship between loss orientation and learning from failure, and that appropriate grieving can promote individual learning behavior. If individual has an overly optimistic attitude toward the failed project too soon after the failure, this is not conducive to learning from the failure. Managers can encourage employees to “get out of the shadow” so they can more effectively from failure.

### Limitations and Future Directions

We recognize that this study has several limitations. First, the data used in this study were cross-sectional, and participants were asked to recall a recent project failure, which may contribute to some biases. In fact, commercial failure often appears random, and it is therefore hard to trace the chain of events that led to the failure. Thus, previous studies usually ask employees to recall such an experience via questionnaires or interviews. To better understand individuals’ reactions after failure, future research should integrate field research and neuroscience-based experiments (Metcalfe, 2017). The key advantage of applying neuroscience methods is to provide more robust conclusions and interpret human behavior from a more fundamental level (i.e., neural processes). Furthermore, in order to further explore the various influencing factors of success and failure in the progress of the project, and further deepen the research, future research can collect data during the project.

Second, the data we collected were from a single point in time and from a single resource (i.e., self-reports of employees). Though we tested Harman’s one factor analysis and CFA to test the risk of CMB, we still recommend that future research use data from multiple sources to make the influence of CMB minimum. We can use coworker or leader reports to see the change of employees’ behavior.

Third, the emotion variable considered in the research model is positive grieving, which is a transitional emotion after an individual experiences a negative event. We only considered one kind of grieving emotion in the study, and did not take into account the mechanism of other emotional variables such as psychological safety (Tjosvold et al., 2004), so future research should also examine other emotional variables as moderators.

Finally, our research considered the role of “mianzi” and other Eastern cultures in the process of learning from failure in the Chinese culture background. Is it possible that there are other Chinese cultural contextual factors that will affect the relationship between individual cognition and behavior? In recent years, many scholars have put forward some Chinese native cultural concepts such as traditionality and Chaxu climate. Are these factors likely to influence individual’s learning process as boundary variables? Additionally, does Western culture have characteristics similar to Chinese contextual factors, and can the research conclusions on Eastern culture be applied to Western culture? Follow-up scholars can further explore from the aspects of cultural differences and commonalities.

## Conclusion

Because failure is a common occurrence in the turbulent world of business, learning from failure is an important research topic. This study validates the effect of a learning goal orientation on learning from failure, and examines the moderating role of positive grieving in the process. We not only enrich the theoretical knowledge of learning from failure, but also provide suggestions on how to promote individual learning after a failure has occurred.

## Data Availability Statement

The raw data supporting the conclusions of this article will be made available by the authors, without undue reservation.

## Ethics Statement

Written informed consent was obtained from the individual(s) for the publication of any potentially identifiable images or data included in this article.

## Author Contributions

WW and SS substantially contributed to the conception and the design of the work as well as in the analysis and interpretation of the data. XC and WY prepared the draft and reviewed it critically. All the authors contributed to the article and approved the submitted version.

## Conflict of Interest

The authors declare that the research was conducted in the absence of any commercial or financial relationships that could be construed as a potential conflict of interest.

## Footnotes

1 This index refers to the moderated mediation effect of the path. If its interval does not contain 0, the moderated mediation effect is significant. What needs to be noted here is the moderator only affects this path.

## Appendix

**APPENDIX TABLE 1:** Hierarchical regression analysis in Stata.

	Restoration Orientation		Loss Orientation		Learning from Failure						
			
Variables	Model 1.1	Model 1.2	Model 2.1	Model 2.2	Model 3.1	Model 3.2	Model 3.3	Model 3.4	Model 3.5	Model 3.6	Model 3.7
Gender	0.083	0.129*	0.164*	0.186*	0.110*	0.138*	0.032	–0.014	–0.017	0.001	0.016
Age	0.015	0.014	0.015	0.015	0.000	0.000	–0.007	–0.010	–0.009	–0.007	–0.008
Education	–0.069	–0.053	−0.206**	−0.192***	–0.025	0.000	0.074*	0.053	0.051	0.053	0.058
Years in the firm	–0.130	–0.123	–0.094	–0.079	–0.045	0.002	0.061	0.085	0.082	0.073	0.076
Years in the team	–0.005	–0.007	–0.008	–0.016	–0.006	–0.019	–0.033	–0.031	–0.031	–0.037	–0.040
Position	–0.010	–0.007	–0.004	–0.001	–0.011	–0.005	0.001	0.000	0.000	0.000	0.000
Type of R&D work	0.050	0.026	0.008	–0.005	0.112**	0.088**	0.073**	0.039	0.037	0.028	0.029
Reason for project failure	0.084	0.063	0.080**	0.082**	–0.058	–0.045	–0.071	–0.045	–0.046	–0.044	–0.043
Learning goal orientation		0.150**		0.132**		0.322***	0.254***	0.164***	0.165***	0.163***	0.159***
Performance-approach goal orientation		0.025		0.084		0.072	0.022	0.025	0.027	0.033	0.032
Performance-avoidance goal orientation		0.168**		–0.001		–0.069	–0.097	–0.018	–0.019	–0.028	–0.030
Restoration orientation							0.126**	0.108*	0.105*	0.094*	0.098*
Loss orientation							0.253**	0.232***	0.230**	0.230***	0.234***
Oscillation orientation							0.206***	0.138***	0.136***	0.129***	0.131***
Positive grieving								0.306***	0.300***	0.289***	0.300***
Negative grieving								−0.168***	−0.169***	−0.164***	−0.158***
Restoration orientation^∗^Positive grieving									–0.016		0.052*
Loss orientation^∗^Positive grieving										−0.080*	−0.115**
*R*^2^	0.019	0.066	0.036	0.073	0.016	0.158	0.367	0.458	0.458	0.468	0.471
△*R*^2^		0.047		0.037		0.142	0.209	0.091	0.001	0.010	0.003
*F*	7.50	15.49	8.61	17.31	5.65	74.94	196.18	480.00	504.83	386.65	400.47
*P*	0.000	0.000	0.000	0.000	0.001	0.000	0.000	0.000	0.000	0.000	0.000

**APPENDIX TABLE 2:** Hierarchical regression analysis without oscillation orientation.

	Restoration Orientation		Loss Orientation		Learning from Failure						
			
Variables	Model 1.1	Model 1.2	Model 2.1	Model 2.2	Model 3.1	Model 3.2	Model 3.3	Model 3.4	Model 3.5	Model 3.6	Model 3.7
Gender	0.083	0.129	0.166	0.189*	0.111	0.138	0.054	0.000	–0.003	0.015	0.029
Age	0.015	0.013	0.014	0.014	0.000	0.000	–0.007	–0.010	–0.010	–0.007	–0.008
Education	–0.070	–0.053	−0.203***	−0.188**	–0.025	0.000	0.065	0.046	0.044	0.047	0.052
Years in the firm	–0.130	–0.124	–0.095	–0.079	–0.044	0.002	0.055	0.081	0.078	0.069	0.071
Years in the team	–0.005	–0.006	–0.009	–0.016	–0.006	–0.019	–0.013	–0.015	–0.016	–0.023	–0.026
Position	–0.010	–0.007	–0.003	–0.001	–0.011	–0.005	–0.003	–0.002	–0.003	–0.003	–0.003
Type of R&D work	0.050	0.036	0.009	–0.005	0.112*	0.088	0.080	0.041	0.039	0.029	0.030
Reason for project failure	0.084*	0.063	0.080	0.082*	–0.058	–0.044	−0.083**	–0.054	–0.054	–0.052	–0.050
Learning goal orientation		0.150***		0.132**		0.322***	0.248***	0.156***	0.158***	0.156***	0.151***
Performance-approach goal orientation		0.025		0.084		0.072	0.042	0.039	0.041	0.047	0.046
Performance-avoidance goal orientation		0.168***		–0.001		–0.069	−0.111**	–0.023	–0.024	–0.033	–0.035
Restoration orientation							0.250***	0.184***	0.180***	0.165***	0.169***
Loss orientation							0.275***	0.242***	0.240***	0.239***	0.243***
Positive grieving								0.330***	0.323***	0.310***	0.321***
Negative grieving								−0.166***	−0.167***	−0.161***	−0.156***
Restoration orientation^∗^Positive grieving									–0.020		0.050
Loss orientation^∗^Positive grieving										−0.084***	−0.118***
*R*^2^	0.008	0.052	0.025	0.058	0.005	0.145	0.332	0.436	0.436	0.447	0.449
△*R*^2^		0.044		0.033		0.14	0.187	0.104	0	0.011	0.002
*F*	1.720	4.622	3.308	5.097	1.484	12.228	28.924	38.615	36.254	37.833	35.955
P	0.090	0.000	0.001	0.000	0.159	0.000	0.000	0.000	0.000	0.000	0.000
